# Global overview of usable Landsat and Sentinel-2 data for 1982–2023

**DOI:** 10.1016/j.dib.2024.111054

**Published:** 2024-10-21

**Authors:** Katarzyna Ewa Lewińska, Stefan Ernst, David Frantz, Ulf Leser, Patrick Hostert

**Affiliations:** a Geography Department, Humboldt-Universität zu Berlin, Unter den Linden 6, 10099 Berlin, Germany; b SILVIS Lab, Department of Forest and Wildlife Ecology, University of Wisconsin-Madison, 1630 Linden Drive, Madison WI 53706, USA; c Geoinformatics –Spatial Data Science, Trier University, Behringstraße 21, Trier 54296, Germany; d Department of Mathematics and Computer Science, Humboldt-Universität zu Berlin, Unter den Linden 6, 10099 Berlin, Germany; e Integrative Research Institute on Transformations of Human-Environment Systems (IRI THESys), Humboldt-Universität zu Berlin, Unter den Linden 6, 10099 Berlin, Germany

**Keywords:** Data availability, Aggregation, Long-term analyses, Terrestrial, Vegetation, Time series

## Abstract

Landsat and Sentinel-2 acquisitions are among the most widely used medium-resolution optical data adopted for terrestrial vegetation applications, such as land cover and land use mapping, vegetation condition and phenology monitoring, and disturbance and change mapping. When combined, both data archives provide over 40 years, and counting, of continuous and consistent observations. Although the spatio-temporal availability of both data archives is well-known at the scene level, information on the actual availability of cloud-, snow-, and shade-free observations at the pixel level is lacking and should be explored individually for each study to correctly parametrize subsequent analyses. However, data exploration is time- and resource-consuming, thus is rarely performed a-priori. Consequently, the spatio-temporal heterogeneity of usable data is often inadequately accounted for in the analysis design, risking ill-advised selection of algorithms and hypotheses, and thus inferior quality of final results. Here we present precomputed data on the daily 1982-2023 availability of usable Landsat and Sentinel-2 acquisitions across the globe. We assembled the dataset by sampling individual pixels at regular intervals with 0.18° spacing in the latitudinal and longitudinal directions and reporting the data availability across the complete time depth of Landsat and Sentinel-2 data archives. The dataset comprises separate Landsat- and Sentinel-2-specific data records. To facilitate data exploration the data availability records are accompanied by a growing season information, also sampled at the pixel-level in regular intervals with 0.18° spacing. The dataset was derived based on freely available 1982–2023 Landsat surface reflectance (Collection 2) and Sentinel-2 top-of-the-atmosphere reflectance (pre-Collection-1 and Collection-1) scenes from 2015 through 2023, following the methodology developed in the recent study on data availability over Europe [1]. Growing season information was derived based on 2001-2019 time series of the yearly 500 m MODIS land cover dynamics product (MCD12Q2; Collection 6) [1]. As such, the dataset presents a unique overview of the spatio-temporal availability of usable daily Landsat and Sentinel-2 data at the global scale, hence offering much-needed a-priori information aiding identification of appropriate methods and challenges for terrestrial vegetation analyses at the local to global scale.

Specifications Table

**Table:** 

Subject	Remote Sensing; Earth-Surface Processes; Big Data Analytics.
Specific subject area	Pixel-level global overview of available of cloud-, snow-, and shade-free Landsat and Sentinel-2 observations for terrestrial vegetation analyses
Data format	Analyzed
Type of data	Tabulated data distributed as .csv
Data collection	We based our dataset on satellite data available freely and openly in the public domain. See the section on *Data source location*. For each scene, we applied commonly used approaches to masked pixels containing cloud, snow, and shade (See the section *Experimental Design and Methods*). The cloud-, snow-, and shade-masked satellite acquisitions were spatially subsampled at the pixel level at the satellite-specific native resolution (i.e., 30 m and 10 m for Landsat and Sentinel-2, respectively) using a regular interval of 0.18°applied in the latitudinal and longitudinal direction as defined in the EPSG:4326 projection. For each sampled pixel we recorded whether a usable value was available on a daily basis (0 – no usable observation; 1 - usable observation). Such systematic sampling design ensures a good representation of natural phenomena and eliminates the clustering of points, which could lead to the underrepresentation of some regions. It is a commonly used approach for deriving statistics for big datasets.
Data source location	Landsat data (Collection 2, doi:10.5066/P918ROHC [2], doi:10.5066/P9TU80IG [3], doi:10.5066/P975CC9B [4]) are freely and openly available in the public domain. We accessed Landsat reflectance Level 2, Tier 1 scenes acquired from 1982 through 2023 through Google Earth Engine in December 2022 – January 2023 and January-February 2024.
	Sentinel-2 data (pre-Collection-1 doi:10.5270/S2_-d8we2fl [5], and Collection-1 doi:10.5270/S2_-742ikth [6]) are freely and openly available in the public domain. We accessed Sentinel-2 top-of-atmosphere (TOA) reflectance Level-1C scenes acquired between 26 June 2015 and 31 December 2023 through Google Earth Engine in July – November 2023 and January-February 2024. MODIS land cover dynamics product at 500-m resolution (MCD12Q2; Collection 6 doi: 10.5067/MODIS/MCD12Q2.061) is freely and openly available in the public domain. We accessed the 2001–2019 time series of data through Google Earth Engine in July 2023.
Data accessibility	Tabular data on 1982–2023 global availability of usable Landsat and Sentinel-2 observations, accompanied by growing season information are publicly available for download in a data repository: Repository name: Dryad Data identification number: 10.5061/dryad.gb5mkkwxm Direct URL to data: https://doi.org/10.5061/dryad.gb5mkkwxm The rasterized version of the tabular data on 1982–2023 global data availability based on Landsat and Sentinel-2 archives can be interactively viewed via Google Earth Engine App: https://katarzynaelewinska.users.earthengine.app/view/worlddataaval Figure 1 was generated based on the tabular data on 1982–2023 global availability of usable Landsat and Sentinel-2 observations using a code available in the GitHub repository https://github.com/kelewinska/Global-Data-Availability-data-in-Brief. The said GitHub repository comprises also a codes executed to generate Fig. 2, as well as examples on how to cast the tabulated data as a georeferenced raster (GeoTIFF) in the EPSG:4326 projection.

## 1 Value of the Data

**Fig. 1:**
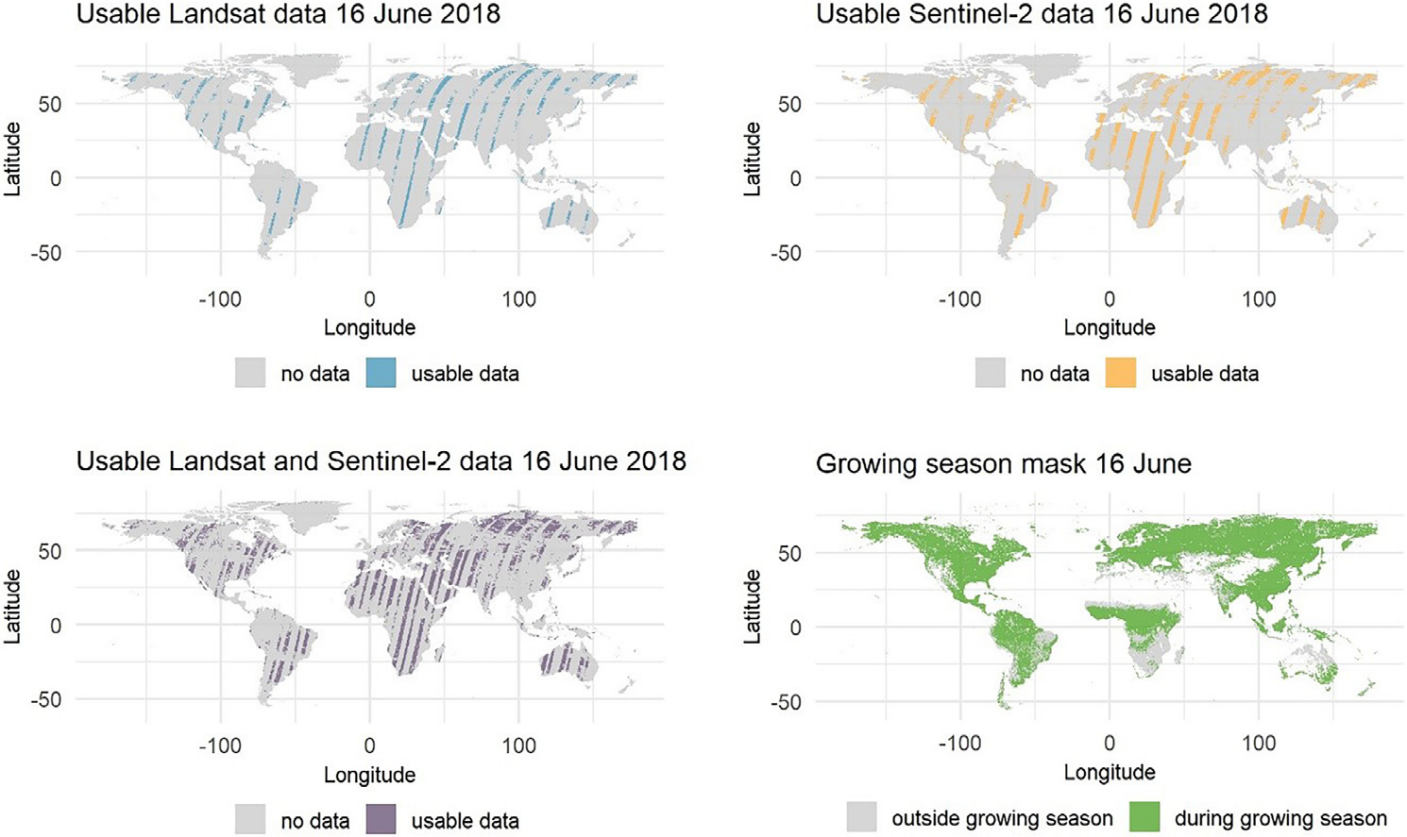
Global availability of usable Landsat and Sentinel-2 data. Example for the 16th June 2018 alongside the respective growing season mask. The figure was generated based on the dataset described in the manuscript using a code available in the GitHub repository https://github.com/kelewinska/Global-Data-Availability-data-in-Brief.

• Understanding data availability is crucial for the appropriate selection and parametrization of algorithms used for terrestrial vegetation analyses. Yet, a-priori data exploration is rarely performed due to its high resource and time requirements. The lack of appropriate understanding of data availability can lead to ill-advised selection of algorithms and poorly framed research hypotheses, and thus inferior quality of the final results. Our dataset provides a ready-to-use, pixel-level global overview of the spatio-temporal availability of cloud-, snow-, and shade-free Landsat and Sentinel-2 observations from 1982 through 2023, allowing for informed decision-making for analyses relying on datasets based on these two data archives.
• The dataset comprises information on the global availability of cloud-, snow-, and shade-free Landsat and Sentinel-2 pixels sampled daily for 1984–2023 using a regular interval of 0.18° applied in the latitudinal and longitudinal directions-. Consequently, a user can easily query data availability for their specific area of interest and time window. As such, the dataset facilitates parametrization of time series processing algorithms, selection of optimal length of compositing windows, evaluation of data availability for spectral–temporal metrics, land cover classification, trend analyses, and other analysis specific to terrestrial vegetation.
• The dataset provides separately availability information for the Landsat (1982–2023) and Sentinel-2 (2015–2023) data archives. The corresponding structure of the two tabulated files comprising the data allows for seamless integration, while catering to users utilizing only one of the data archives. Furthermore, this separation allows for a straightforward assessment of the added value of joint use of Landsat and Sentinel-2 archives after 2015, as compared to Landsat or Sentinel-2 time series alone.
• The pre-calculated overview of usable data provides insight into the quality of formerly derived datasets and results based on Landsat and/or Sentinel-2 time series that lack explicit data-availability quality assessment.
• The accompanying Google Earth Engine App (https://katarzynaelewinska.users.earthengine.app/view/worlddataaval) offers on-the-fly querying of the datasets. Provided functionality allows exploration of the data availability for a selected sensor constellation, using a user-defined length of aggregation period, and allowing to choose an entire calendar year, a vegetation-specific growing season, or other user-defined time periods. As such, the App provides an interface with a basic data query functionality for exploring Landsat and Sentinel-2 data availability that is designed to be used by a wide range of user groups (Fig. 1).

## 2 Background

While developing analysis workflows based on Landsat and Sentinel-2 time series, a-priori information on data availability on annual and multi-annual basis is needed to properly parametrize algorithms for vegetation analysis. Often, parametrization choices are made based on educated guesses, trial-and-error, or ‘expert knowledge’ of the availability of satellite data acquisitions. However, many regions are prone to frequent cloud and snow cover, and different observation capacities due to limited download or on-board storage limitations. These factors inflict lower availability of usable data comparing with the theoretically possible availability arising from data acquisition frequency. Specifically, inexperienced users often struggle to find a suitable parameterization of their analysis workflows. Recognizing the existing information gap, we present a global 1982–2023 overview of cloud-, snow-, and shade-free Landsat and Sentinel-2 observations sampled at a pixel-level at a regular 0.18° interval [7]. The dataset provides a readily available overview of the usable data coverage, thus supporting, for example, the informed selection of algorithms and compositing windows, and aiding the parametrization of specific vegetation-focused analyses. The dataset builds upon our previous study on the availability of usable Landsat and Sentinel-2 data over Europe [1], now providing global coverage and extending the time series through 2023.

## 3 Data Description

The article describes the dataset in the linked repository, which comprises the 1982–2023 global overview of daily availability of cloud-, snow-, and shade- free Landsat and Sentinel-2 observations [7]. The data were sampled for individual land pixel using a regular 0.18° interval defined in the EPSG:4326 projection and applied in the latitudinal and longitudinal directions, and span area between -179.8867°W and 179.5733°E and -59.05167°S and 83.50834°N, totalling 475,150 sample points. Each sample comprises daily data availability (binary information on usable data/no usable data) sampled from a single pixel at a regularly distributed sampling location. The complete dataset in the linked repository consists of three files comprising pixel-level daily data availability information for i) 1982-2023 Landsat and ii) 2015–2023 Sentinel-2 time series, as well as iii) auxiliary growing season information distributed as a mask in two variants, i.e., for a regular and a leap year (Table 1). The growing season information was also sampled using the same regular 0.18° sampling interval as Landsat and Sentinel-2 time series.

**Table 1:** Datasets shared through the linked repository [7].

File name	Explanation
GLOBAL_LND_1982–2023_CSO.csv	Daily data availability derived from Landsat 1982–2023 archives
GLOBAL_S2_2015–2022_CSO.csv	Daily data availability derived from Sentinel-2 2015–2023 archives
GLOBAL_GrowingSeason.csv	Growing season information for normal and leap years
README.md	Text file containing basic information on the distributed datasets

Each dataset is distributed in a tabulated format (.csv) and consists of 475,150 observations representing the global regularly distributed sample points. Each observation is characterized by a unique identifier and coordinates (Table 2). The binary information on availability of cloud-, snow-, and shade-free observation (i.e., 1 – valid observation; 0 – no valid observation) is given for the *GLOBAL_LND_1982–2023_CSO.csv* and *GLOBAL_S2_2015–2023_CSO.csv* files on a daily basis in variables named *L_YYYY_MM_dd* (Table 2). For the Landsat-specific dataset, the valid range of dates is 1982–08–22 through 2023–12–31, while for Sentinel-2-specific dataset the valid range of dates is 23-06–2015 through 2023–12–31. The auxiliary dataset containing information on growing season consist of two sets of variables providing daily growing season masks. The first set of variables is specific to a regular year, whereas the second set characterizes a leap year (Table 2).

**Table 2:** Overview of variables available in each dataset distributed through the linked repository.

Variable	Explanation
All datasets	
Id	Unique identifier
Lat	Latitude [in degrees] (EPSG:4326)
Lon	Longitude [in degrees] (EPSG:4326)
GLOBAL_LND_1982–2023_CSO.csv	
L_YYYY_MM_dd	Data availability (binary information: 1 – valid observation; 0 – no valid observation) for a single day where YYYY indicates year, MM indicates month, and dd indicates day.
GLOBAL_S2_2015–2023_CSO.csv	
L_YYYY_MM_dd	Data availability (binary information: 1 – valid observation; 0 – no valid observation) for a single day where YYYY indicates year, MM indicates month, and dd indicates day.
GLOBAL_GrowingSeason.csv	
Regular_MM_dd	Information on growing season (1 – within the growing season, 0 – outside the growing season) provided daily for a regular year comprising 365 days, where MM indicates month and dd day of a day of interest.
Leap_MM_dd	Information on growing season (1 – within the growing season, 0 – outside the growing season; NA – no data) provided daily for a leap year comprising 366 days, where MM indicates month and dd day of a day of interest.

The dataset is distributed in .csv format ensuring easy ingestion and facilitating manipulation in scripting languages and data processing software.

Furthermore, the data are also available through the Google Earth Engine App interface (https://katarzynaelewinska.users.earthengine.app/view/worlddataaval), allowing for on-the-fly interactive query based on a set of predefined criteria.

## 4 Experimental Design, Materials and Methods

We based our analyses on freely and openly accessible Landsat and Sentinel-2 data archives available in Google Earth Engine [8]. We used all Landsat surface reflectance Level 2, Tier 1, Collection 2 scenes acquired with the Thematic Mapper (TM) [2], Enhanced Thematic Mapper (ETM+) [3], and Operational Land Imager (OLI) [4] scanners between 22nd August 1982 and 31st December 2023, and Sentinel-2 TOA reflectance Level-1C scenes (pre-Collection-1 [5] and Collection-1 [6]) acquired with the MultiSpectral Instrument (MSI) between 23rd June 2015 and 31st December 2023.

We implemented a conservative pixel-quality screening to identify cloud-, snow-, and shade-free land pixels. For Landsat time series, we relied on the inherent pixel quality bands [9,10] excluding all pixels flagged as cloud, snow or shadow as well as pixels with the fill-in value of 20,000 (scale factor 0.0001; [11]). The accuracy of the inherent pixel quality band distributed with the operational Landsat Collection 2 data varies depending on the Landsat scanner (due to the presence or absence of thermal and cirrus-specific bands) and is characterized by 1–41 % omission and 8–23 % commission. Identification of cloud shadows is performed with 13–26 % omission and 1–5 % commission errors [9]. Due to the Landsat 7 orbit drift [12] we excluded all ETM+ scenes acquired after 31st December 2020.

Because Sentinel-2 Level-2A quality masks lack the desired scope and accuracy [13,14], we resorted to Level-1C scenes that are accompanied by the supporting Cloud Probability product. Furthermore, to identify usable pixels we employed a selection of conditions, and additional analyses, such as Cloud Displacement Index and a threshold on Band 10 (SWIR-Cirrus), which are not accessible at Level-2A. Overall, our Sentinel-2-specific cloud, shadow, and snow screening comprised:

‐ Exclusion of all pixels flagged as clouds and cirrus in the inherent ‘QA60’ cloud mask band (balanced overall accuracy of 58 %, with middle omission error of 50 % and middle commission error of 63 % [15]);.
‐ Exclusion of all pixels with cloud probability >50 % as defined in the corresponding Cloud Probability product available for each scene (The overall accuracy of 85–96 % with omission error of 3–24 % and commission error of 1–14 %, depending on the type of the cloud, land cover type, and the reference dataset [16]. The balanced overall accuracy lies between 79 % and 96 % [15,16]).
‐ Exclusion of cirrus clouds (B10 reflectance >0.01) (omission error 62 %, commission error of 82 %) [17].
‐ Exclusion of clouds based on Cloud Displacement Analysis (CDI<-0.5) (The overall accuracy of 95 %, with omission error of 5 % and commission error of 7 %. Among others, enhances the identification of low-altitude cirrus clouds, but since is based on the Potential Cloud Pixel is therefore prone to commission in the build-up areas [18]).
‐ Exclusion of dark pixels (B8 reflectance <0.16) within cloud shadows modelled for each scene with scene-specific sun parameters for the clouds identified in the previous steps. Here we assumed a cloud height of 2000 m.
‐ Exclusion of pixels within a 40-m buffer (two pixels at 20-m resolution) around each identified cloud and cloud shadow object. Increases reliability of masking on the cloud fringe, but may lead to greater commission over build-up land cover [16].
‐ Exclusion of snow pixels identified with a snow mask branch of the Sen2Cor processor (omission error of 7 % commission error of 53 %) [17].

Through applying the data screening, we generated a time series of daily availability records for Landsat and Sentinel-2 data archives. We next sampled individual pixels in the resulting binary time series spacing them 0.18° apart in the latitudinal and longitudinal directions as defined in the EPSG:4326 projection, obtaining 475,150 sample points located over land between -179.8867°W and 179.5733°E and 83.50834°N and -59.05167°S.

Owing to the substantial amount of data comprised in the Landsat and Sentinel-2 archives and the computationally demanding, particularly for Sentinel-2, process of cloud-, snow-, and shade-screening, we performed the data screening and sampling in batches corresponding to a 4° x 4° regular grid, and consolidated the final data in post-processing.

**Fig. 2:**
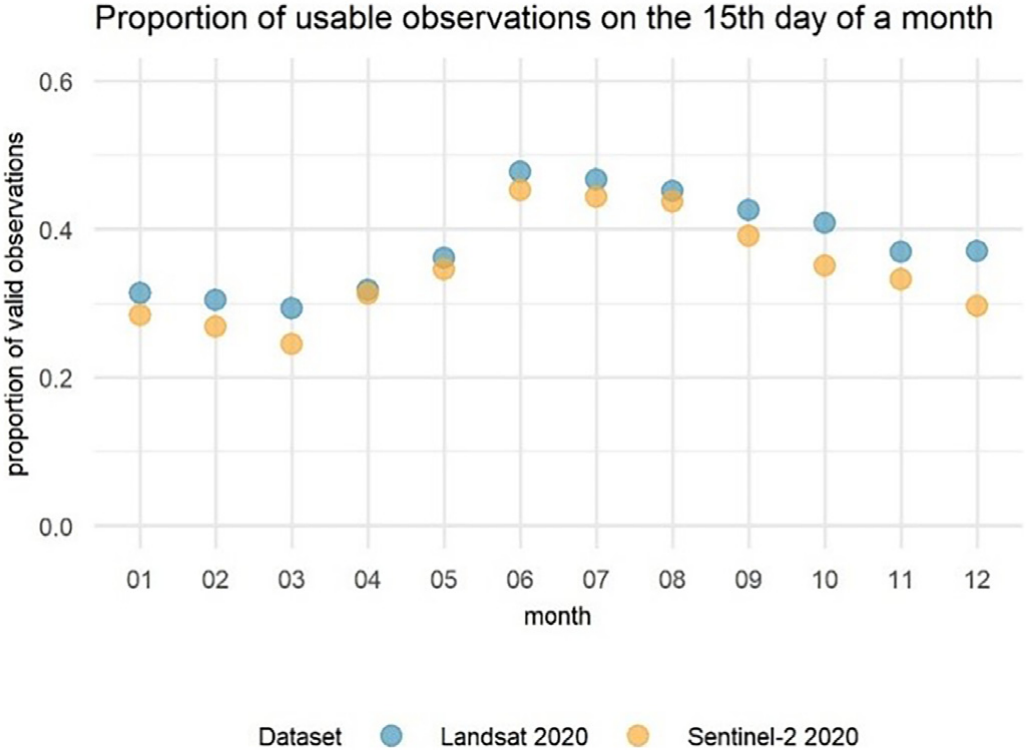
Proportion of valid observations for Landsat and Sentinel-2 on 15th day of each month in 2020. The figure was generated using a code available in the GitHub repository https://github.com/kelewinska/Global-Data-Availability-data-in-Brief.

To check the consistency of the applied cloud-, snow-, and shade-screening between Landsat and Sentinel-2 masking approaches, we compared the proportion of usable observations to all observations acquired on a selection of days, namely on the 15th day of each month in 2020 (Fig. 2). We noted very strong correspondence between the results, which demonstrates that the ensembled of conditions we used for Sentinel-2 performed comparably to the inherent Landsat pixel quality bands. However, it is important to note that Landsat and Sentinel-2 have different swath widths (185 vs. 290 km, respectively) and the overlap between the daily acquisitions is relatively small at the global scale. Consequently, the presented comparison, although informative, does not show the performance of cloud-, snow-, and shade-screening for the same pixels, and the same acquisition time.

We derived the pixel-specific growing season information from 2001 to 2019 time series of the yearly 500-m MODIS land cover dynamics product (MCD12Q2; Collection 6) available in Google Earth Engine. We only used information on the start and the end of a growing season, excluding all pixels with quality below ‘best’. When a pixel went through more than one growing cycle per year, we approximated a growing season as the period between the beginning of the first growing cycle and the end of the last growing cycle. To fill in data gaps arising from low quality data and insufficiently pronounced seasonality [19], we used a 5 × 5 mean moving window filter to ensure better spatial continuity of our growing season datasets. Following [1], we defined the start of the season as the pixel-specific 25th percentile of the 2001–2019 distribution for start of the season dates, and end of the season as the pixel-specific 75th percentile of the 2001–2019 distribution for end of the season dates. Finally, we sampled the start and end of the season datasets using the same approach applied to Landsat and Sentinel-2 time series, i.e., sampling individual pixels spaced 0.18° apart in the latitudinal and longitudinal direction, as defined in the EPSG:4326 projection.

## Limitations

Our dataset relies on the cloud, shadow, and snow masking functionality available in Google Earth Engine. While for Landsat we relied on Fmask version 3.3.1 [9,10], for Sentinel-2 we needed to rely on an ensemble of common approaches. Although all cloud detection algorithms carry a certain level of uncertainty [15,16], and for the Sentinel-2-specific workflow can result in lower performance over build-up areas [13,16,18], our analyses are conservative and provide a valid generic overview.

Since our dataset relies on a systematic sampling of individual pixels located 0.18° apart, the derived availability may not capture some of the local variability in cloud-, snow-, and shade-free Landsat and Sentinel-2 observations. Nevertheless, by following the commonly used in the big data analyses approach of systematic sampling the dataset provides a robust overview of the general patterns at landscape to global scale.

The quality of our auxiliary growing season dataset is sometimes restricted due to, at times, low input data quality and insufficiently pronounced seasonality in the original MODIS time series [19].

## Ethics Statement

Not applicable. Our work does not involve any use of human subjects, animal experiments or data collected from social media platforms.

## CRediT Author Statement

**Katarzyna Ewa Lewińska**: Conceptualization, Methodology, Formal analysis, Investigation, Visualization, Data curation, Writing –original draft, Visualization; **Stefan Ernst**: Data curation, Writing –review & editing; **David Frantz**: Writing –review & editing; **Ulf Leser**: Writing –review & editing, Funding acquisition; **Patrick Hostert**: Supervision, Writing –review & editing, Funding acquisition.

## Data Availability

DryadGlobal overview of cloud-, snow-, and shade-free Landsat (1982–2023) and Sentinel-2 (2015–2023) data (Original data).
